# Carbon nanotube bumps for the flip chip packaging system

**DOI:** 10.1186/1556-276X-7-105

**Published:** 2012-02-07

**Authors:** Chin Chong Yap, Christophe Brun, Dunlin Tan, Hong Li, Edwin Hang Tong Teo, Dominique Baillargeat, Beng Kang Tay

**Affiliations:** 1 CINTRA CNRS/NTU/THALES, UMI 3288, Research Techno Plaza, 50 Nanyang Drive, Border X Block, Level 6, Singapore, 637553, Singapore; 2 School of Electrical and Electronics Engineering, Nanyang Technological University, Block S1, 50 Nanyang Avenue, Singapore, 639798, Singapore; 3 XLIM UMR 6172, Université de Limoges/CNRS, 123 Avenue Albert Thomas, Limoges Cedex, 87060, France; 4 Temasek Laboratories@NTU, Research Techno Plaza, 50 Nanyang Drive, Border X Block, Level 9, Singapore, 637553, Singapore

**Keywords:** CNT bumps, interconnects, flip chip, packaging

## Abstract

Carbon nanotube [CNT] interconnection bump joining methodology has been successfully demonstrated using flip chip test structures with bump pitches smaller than 150 μm. In this study, plasma-enhanced chemical vapor deposition approach is used to grow the CNT bumps onto the Au metallization lines. The CNT bumps on the die substrate are then 'inserted' into the CNT bumps on the carrier substrate to form the electrical connections (interconnection bumps) between each other. The mechanical strength and the concept of reworkable capabilities of the CNT interconnection bumps are investigated. Preliminary electrical characteristics show a linear relationship between current and voltage, suggesting that ohmic contacts are attained.

## Introduction

Flip chip technology is one of the off-chip interconnect methodologies used in electronic packaging. According to the International Technology Roadmap for Semiconductor, the forecasted requirement for flip chip bump pitches will be shrinking them beyond 150 μm [1]. However, traditional solder bumps had difficulties downscaling beyond the 100-μm pitch size due to the high diffusive and softening nature of the solder [2]. Carbon nanotubes [CNTs] show excellent electrical, thermal, and mechanical properties and have been viewed as one of the emerging choices for future flip chip interconnect [3]. As compared with metal, CNTs possess a higher current carrying capacity (10^9 ^A/cm^2^), and theoretical studies had also shown that CNTs have a negligible skin depth effect and are free from the high-frequency current crowding issue due to their large kinetic inductance and negligible magnetic inductance [3]. These advantages motivate the researchers to evaluate the performance of the CNT bump for interconnect usage in both direct current [DC] and high frequency applications [3,4].

CNT bumps had been demonstrated by several groups as potential off-chip interconnects [4-6]. Soga et al. have shown the bumps' good mechanical flexibility and low bundle resistance of 2.3 Ω (for a 100-μm diameter bump) [5]. Hermann et al. demonstrated a reliable electrical flip chip interconnect using CNT bumps over 2,000 temperature cycles [4]. CNT bumps for practical applications such as high-power amplifier application had also been demonstrated [6]. In all the mentioned works, the CNT bumps were grown using the chemical vapor deposition [CVD] approach. The mechanism for vertical alignment during the CVD approach is achieved by the van der Waals forces between the walls of CNTs, resulting in tubes that are not exactly 'aligned' [7]. The poor 'alignment' forms bends, reduces the mean free path [m.f.p.], and increases the resistance of CNTs [8]. Plasma-enhanced CVD [PECVD] is able to resolve this issue by the introduction of electric field to achieve alignment as well as lower growth temperature [9].

In the flip chip scenario, the as-grown CNT bumps are usually pressed onto pre-patterned conductive adhesive [5] or solder materials [10] to form the connections. This approach requires heating up the structure to a minimum of 200°C in order to reflow the materials to obtain good contacts. Yung et al. demonstrated a large-scale assembly process using the CNT interconnection bump showing that CNTs adhere well to each other by van der Waals force interactions [11]. However, no work using the CNT interconnection bump was reported for CNT bump pitches below 150 μm which is a requirement for the future flip chip technology.

For the first time, we demonstrated the CNT interconnection bump joining methodology for pitches smaller than 150 μm. The fabrication methodology can be divided into two parts: (1) the growth of CNT bumps on both sides of the substrate using the PECVD approach and (2) the alignment and 'insertion' of the CNT bumps into each other using a flip chip bonder machine. Moreover, we discuss the technological aspects of developing the test structure and present the initial DC behavior of small-scale CNT interconnection bumps.

## Methods

### Design and fabrication of test structure and CNT bumps

**Figure 1:**
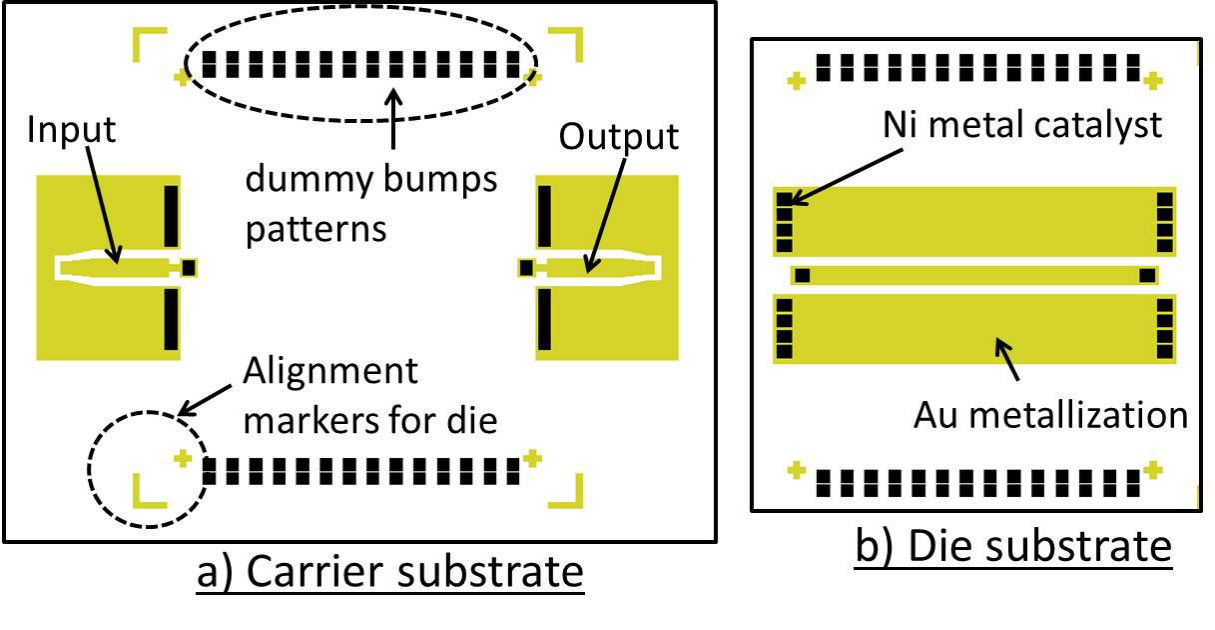
**Schematic of the flip chip test structure used in this experiment**. (**a**) The carrier and (**b**) the die designs. Au metal is used as the metallization material and Ni as the catalyst for the CNT growth. At the sides of each structure, two rows of square patches are also patterned and deposited with the Ni catalyst on Au for CNT growth. These additional CNT bumps serve as dummy bumps to increase bump densities for mechanical supports.

The flip chip test structure used in this experiment consisted of a carrier and a die structure as shown in Figure 1. The dimension of the carrier is approximately 6 × 5 mm, while the die is 4 × 4 mm. The choice of barrier layer to grow CNTs on Au metallization and the detailed fabrication details were described elsewhere [12]. Briefly, e-beam evaporation was used to deposit Au (1 μm) onto what would serve as the metallization layer while a second layer of TiN (50 nm) barrier and Ni (15-nm metal catalyst for CNT growth) were deposited onto the predefined patterns above the Au metallization using a lift-off technique.

**Figure 2:**
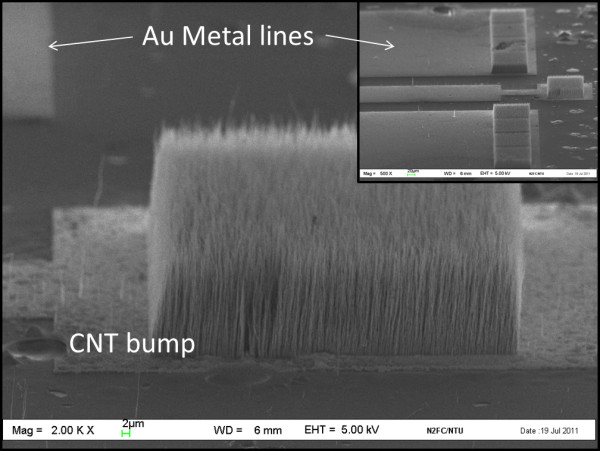
**SEM of a CNT bump growth using the PECVD approach**. The grown CNT was vertically aligned, and the length of the CNT was 20 μm. The top right (inset) shows the low magnification view of the CNT bumps formed on the Au metal lines.

The wafers were diced to their specified dimensions and cleaned in deionized water before they were transferred to a PECVD chamber where the CNT growth will be performed. The CNT growth was conducted at 750°C with a plasma power of 85 W for 30 min. The growth pressure was at 6 mbars with a gas ratio of 1:5 (C_2_H_2_/NH_3_). CNT bumps with a height of approximately 20 μm were obtained as shown in Figure 2. Each of these CNT bumps comprised multiwall CNTs [MWCNTs] with an average diameter of around 100 nm. Subsequently, a Panasonic flip chip bonder machine (Panasonic Factory Solutions Company, Rolling Meadows, IL, USA) was use to perform the die alignment and attachment. A load setting of 0.5 kg with a bonding time of 30 s was used as the bonding parameter. No bonding temperature was used in our experiment.

In order to compensate for the alignment accuracy of +/-10 μm of the bonder machine, the CNT bumps on the metallization lines were deliberately fabricated to be rectangular. For example, the size of one bump on the carrier is 120 × 100 μm, while that on the die is 100 × 120 μm. This enlarged bump area helped to decrease the occurrence of open circuitry and reduced the connection resistance due to misalignment [13]. In order to improve the mechanical support between CNTs and metal contacts, nonconducting adhesives are usually employed [4]. In contrast, we introduced rows of dummy CNT bumps positioned at the sides of the chip and die to increase the densities of CNT bumps. The dummy bumps provided additional mechanical support to hold the weight and leveled the attached die.

Another area of concern is that the growth temperature of CNTs used in this work is higher than that in the typical CMOS backend process (400°C) [14]. Lowering the growth temperature of the CNTs increased the defects and decreased the degree of crystallinity of the CNT structure, which affected the electrical characteristics of CNTs [15,16]. Improving the quality of the CNTs at low temperature is still under investigation, and the variation of metal resistance before and after the CNT growth process will be taken into consideration. In our previous work, the change in Au metal line resistance was found to be insignificant as the Au resistance is smaller than the measured CNT bump resistance, as reported in the latter part of this report [12].

## Results and discussion

**Figure 3:**
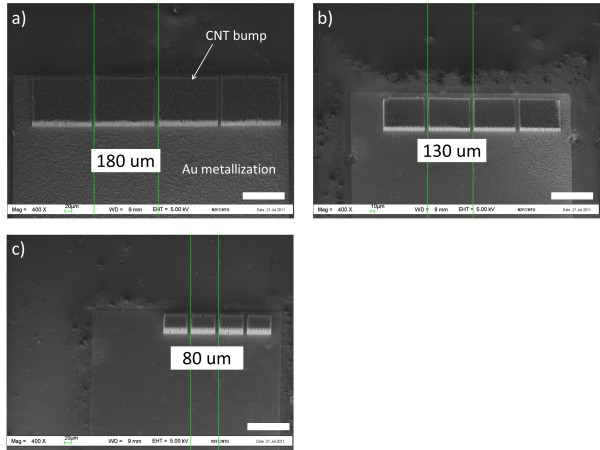
**Illustration of CNT bumps on the Au electrode with three different pitches**. The smallest designed pitch is 80 μm in this work. The dimensions of each CNT bump are (**a**) 170 × 150 μm for structure 1, (**b**) 120 × 100 μm for structure 2, and (**c**) 70 × 50 μm for structure 3. The scale bar at the bottom right of each image represents 100 μm.

To demonstrate the feasibility of using PECVD approaches to achieve fine pitch CNT bumps, three different sets of test structures comprising (structure 1) 170 × 150 μm, (structure 2) 120 × 100 μm, and (structure 3) 70 × 50 μm CNT bump sizes were designed (Figure 3). The scanning electron microscopy [SEM] images in Figure 3 show the CNT bumps grown using the PECVD approach which allowed pitches to downsize to 80 μm in our experiment. A homogenous CNT bump height can also be observed throughout the carrier and die (dummy and CNT bumps on electrodes). Larger catalyst pattern geometry would result in longer CNT length due to the differences in partial pressure of carbon feedstock gas [17]. The effect of catalyst pattern geometry was not significant in this experiment, and the height of all CNT bumps was assumed to be 20 μm regardless of the bumps' dimensions.

**Figure 4:**
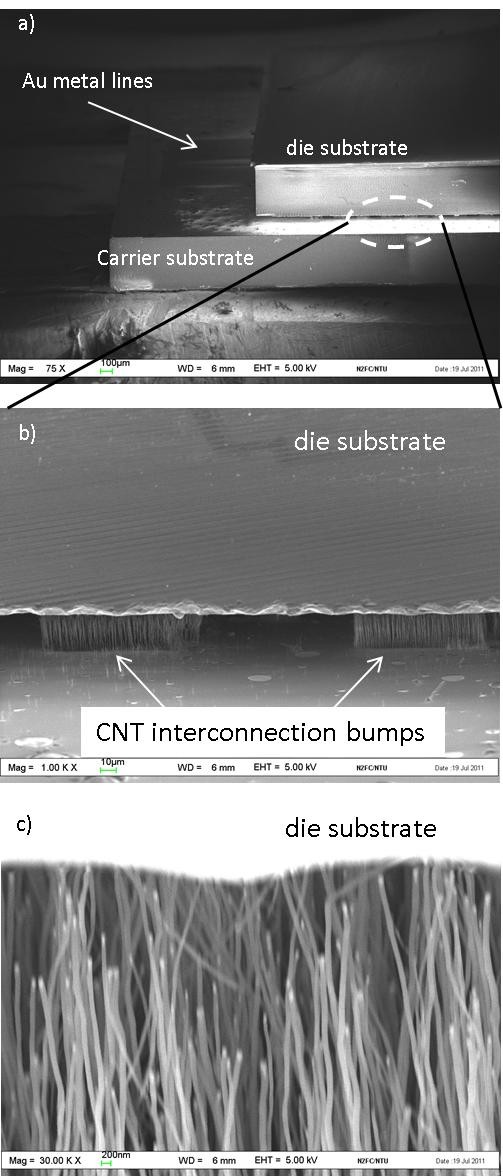
**SEM images of the CNT interconnection bump demonstrated using a flip chip concept**. The dimensions of CNT bumps were 100 × 100 μm. (**a**) Die attached to the carrier at a tilted angle of 75°. (**b**) Magnified view of two CNT interconnection bumps. (**c**) CNT from the bottom carrier was observed to be touching the die substrate which is indicative of the connections made.

For the first time, CNT interconnection bump joining methodology for fine pitch bump had been achieved, as depicted in Figure 4. In Figure 4a, the flip chip test structure was observed at an angle of 75° under the SEM. A gap of approximately 20 μm, which is equivalent to the CNT height, can be observed in Figure 4b. The bonding load of 0.5 kg was sufficient to cause the CNTs from the bottom carrier to 'insert' and touch the top die, as observed in Figure 4c. The load of 0.5 kg, which is equivalent to 4.5 N or 3.125 kg/cm^2^, is much lower than those applied in previous reported flip chip experiments [4-6]. In this joining methodology, the CNT bumps are 'inserted' through the air gaps of opposite CNT bumps thus requiring smaller force. Due to equipment limitations, microphotographs of the CNT bumps during the bonding and release processes could not be captured to demonstrate the mechanical flexibility of CNT bumps as observed by Soga et al. [5]. However, based on the SEM images in Figure 4b, the vertical alignment of the CNT bumps can still be observed, which is likely due to the mechanical flexibility of the CNT bumps.

In this experiment, structures 1 and 2 were tilted to 75° in the SEM to observe the gap between test structures. This is remarkable as no bonding temperature or adhesive was used during this bonding process to mechanically bond the die to the carrier. However, for structure 3, the die tended to slide off from the carrier during movement of the SEM's stage. The dummy bumps in structures 1 and 2 were effective in holding the weight of the die (0.033 g), but not in structure 3. Assuming a perfect alignment, the area occupied by a total of 74 CNT bumps on structure 2 is 0.74 mm^2 ^out of 16 mm^2 ^(die area). On the other hand, the area occupied by CNT bumps in structure 3 is 0.185 mm^2 ^out of 16 mm^2^. This is logical because the dummy bumps in structure 3 were smaller as compared with those in structures 1 and 2. Based on structure 3, future designs using the die area will require more than 1.2% to be converted for the implementation of the CNT bump area for sufficient mechanical support. This is to ensure that the bonding forces present within the CNT interconnection bumps is greater than the die's weight to ensure mechanical stability. A study based on total energy and molecular dynamic calculation on molecular CNT to CNT joining methodology shows that the type of bonding present between CNTs is very strong, and a force larger than 3 nN will be required to disengage the joining structure [18]. Yung et al. suggested the van der Waals forces between CNTs to be the bonding mechanism when CNTs were inserted into each other [11]. In this work, only the dimensions of bumps (area) were varied; the influence of densities and diameter of CNTs within a bump may also be an important consideration to CNT bonding mechanisms.

**Figure 5:**
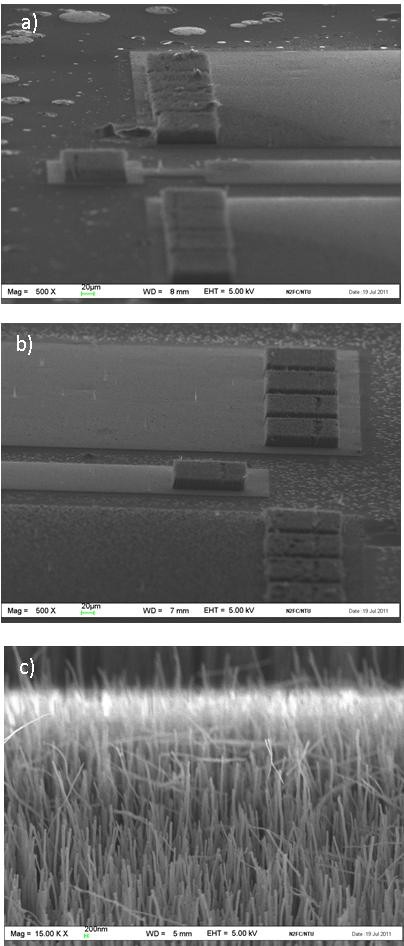
**SEM images of the CNT bumps' morphology after top die removal**. (**a**) CNT bumps on the carrier. (**b**) CNT bumps on the chip joined to the carrier bump in (a). (**c**) Magnified view of the bump. The vertical alignments of the CNTs are indications of the mechanical flexibility of CNTs.

The attached die was subsequently removed from the carrier using tweezers to observe the effect of the CNT bumps after bonding. The carrier and die were then loaded into the SEM chamber with the same orientation to observe the conditions of CNT bumps that were in contact with each other, as shown in Figure 5. A portion of the CNT bump appeared to be smeared as seen from Figure 5a, but a high percentage of CNT bumps retained their original structure. This is similar to the observations of Yung et al. for large-scale CNT to CNT interconnect structure which demonstrated that the bonding process is reworkable [11].

**Figure 6:**
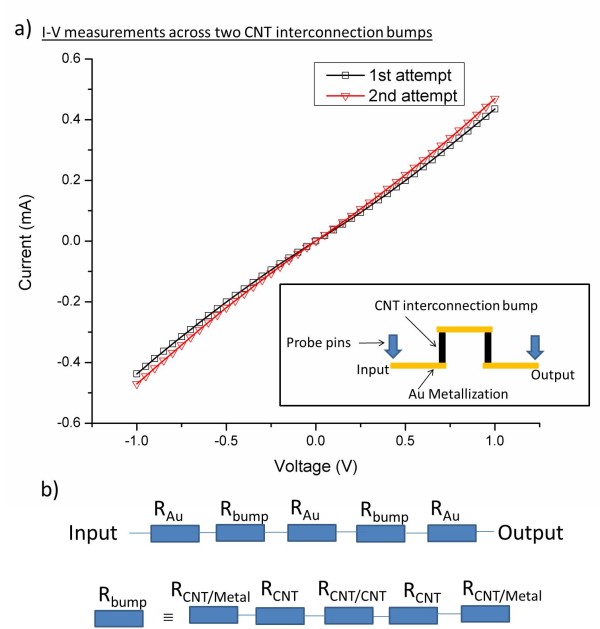
**Two-point probe DC measurement across two interconnection bumps measured across the carrier substrate's input and output**. The CNT bump dimensions were 150 × 150 μm. (**a**) Similar *I*-*V *characteristics were observed to demonstrate the concept of reworkable process using this form of joining methodology. (**b**) Equivalent electrical circuit for the measured resistances and CNT interconnection bump.

To verify the CNT interconnection bundle resistances as well as the concept of reworkable capabilities of the joining methodology, the die of test structure 1 was removed and was bonded to the same carrier again to measure the *I*-*V *characteristics. Using a two-point probe, the DC measurement was performed across the input and output nodes (two CNT interconnection bumps) as described in Figure 1. *I*-*V *measurements in Figure 6 displayed a similar DC behavior which demonstrated the concept of a reworkable process. The slight deviation of the second attempt could be caused by the differences in die placement due to the limited alignment accuracy of the bonding machine. The current increased linearly with the voltage applied, indicating that ohmic contact has been achieved between CNTs and metallization [19]. The line of the 'first attempt' was fitted linearly to obtain a slope of 4.20 × 10^-4 ^A/V. This indicates that a CNT interconnection bundle resistance of 1,190 Ω was achieved.

The equivalent circuit of the CNT interconnection bundle resistance is represented in Figure 6b, and three major resistance components account for the observed *I*-*V *characteristic: metal-to-CNT resistance [*R*_CNT/metal_], CNT intrinsic resistance [*R*_CNT_], and CNT-to-CNT interface resistance [*R*_CNT/CNT_]. Since the CNTs were grown directly on metallization, the type of bonding formed between CNTs and the metal is strong, and *R*_CNT/metal _is insignificant and can be neglected [20]. The m.f.p. of MWCNTs is in the order of approximately 25 μm, and *R*_CNT _scales with the length above the m.f.p. [3]. The length of our MWCNTs is less than the m.f.p. and does not account for the differences in the CNT bump resistances as seen across various devices (not shown). As such, the only possible reason for the measured resistance is likely to be cause by *R*_CNT/CNT_.

*R*_CNT/CNT _is affected by the chirality of CNTs, the interface area, the gap between CNTs, and the interface imperfections [21,22]. It is now common knowledge that the properties of the CNTs within the CNT bump cannot be identical and that each CNT varies in terms of diameters and chirality. However, the work functions for CNTs are identical as they share the same graphene band structure [21]. For MWCNTs, majority of the current only passes through the outermost layer of MWCNTs, and a metallic behavior is expected [23]. Even if the MWCNT is not metallic, the bandgap of semiconducting CNTs can be estimated as *E*g = 0.9 eV/diameter, approximating a bandgap of 9 meV for a CNT diameter of 100 nm [16]. In this case, the Fermi level of the CNT in contact with another CNT will align within the bandgap of the semiconducting CNT. The resulting band structures have a negligible Schottky barrier height due to the small bandgap. Thus, the metal-to-CNT and CNT-to-CNT interfaces are similar to the metal-to-metal junctions. The major transport mechanism for electron transport is through tunneling across atomic thick air gap, resulting in an ohmic and linear *I*-*V *behavior observed in Figure 6a[22].

From the literature, a typical resistance between individual metallic CNT to metallic CNT separated by van der Waals distance of 0.34 nm is 200 kΩ [21]. In our work, the gap may be much larger than 0.34 nm, resulting in a huge tunneling resistance through the air gap and absorbents [24]. Nevertheless, the results obtained from other experiments involving CNT-to-CNT contacts displayed positive results and outlook for use as alternatives to solve the high contact resistance between the CNT tip and metal [11,20]. More work will be required to study the formation of reliable and high-performance small-scale CNT interconnection bumps for use as vertical interconnects in the future.

## Conclusion

The feasibility to use the PECVD approach to achieve CNT bump pitches smaller than 150 μm has been studied. The introduction of the TiN barrier layer between the Ni catalyst and Au metallization allows an efficient growth of CNT bumps directly on Au while maintaining good electrical connections between the CNT and Au electrodes. The successful growth of CNTs on Au metallization opens up opportunities to evaluate the performance of vertically aligned CNT bundles in very high frequency domain. By increasing the densities of CNT bumps, the first demonstration of CNT-to-CNT joining methodology with bump pitches smaller than 150 μm had been successful. The preferred densities of CNT bumps (area) to the die area must be higher than 1.2% to achieve sufficient mechanical support. Preliminary DC characterization suggests an ohmic contact behavior with a CNT interconnection bundle resistance of 1 kΩ. Most importantly, the CNT bumps were not damaged after repeated bonding, and similar *I*-*V *measurement had been achieved.

## Competing interests

The authors declare that they have no competing interests.

## Authors' contributions

CCY and DT carried out the fabrication and the characterization of the structures, as well as drafted the manuscript. CB carried out the simulation and proposed the design. HL and EHTT participated in the analysis and discussion of the results. BKT and DB conceived the study and participated in its design and coordination of the team. All authors read and approved the final manuscript.

## References

[B1] International Technology Roadmap for Semiconductors 2009 Edition Assembly and Packaging http://www.itrs.net/Links/2009ITRS/2009Chapters_2009Tables/2009_Assembly.pdf

[B2] Tummala R Wong CP Markondeya Raj P Nanopackaging research at Georgia Tech Nanotechnol Mag, IEEE 2009 3 20 25

[B3] Hong L Chuan X Srivastava N Banerjee K Carbon nanomaterials for next-generation interconnects and passives: physics, status, and prospects Electron Devices, IEEE Trans on 2009 56 1799 1821

[B4] Hermann S Pahl B Ecke R Schulz SE Gessner T Carbon nanotubes for nanoscale low temperature flip chip connections Microelectron Eng 2010 87 438 442 10.1016/j.mee.2009.05.027

[B5] Soga I Kondo D Yamaguchi Y Iwai T Mizukoshi M Awano Y Yube K Fujii T Carbon nanotube bumps for LSI interconnect58th Electronic Components and Technology Conference: 27-30 May 2008; Florida 2008 New York: IEEE 1390 1394

[B6] Iwai T Shioya H Kondo D Hirose S Kawabata A Sato S Nihei M Kikkawa T Joshin K Awano Y Yokoyama N Thermal and source bumps utilizing carbon nanotubes for flip-chip high power amplifiers Electron Devices Meeting, 2005 IEDM Technical Digest IEEE International 2005 New York: IEEE 257 260

[B7] Fan S Chapline MG Franklin NR Tombler TW Cassell AM Dai H Self-oriented regular arrays of carbon nanotubes and their field emission properties Science 1999 283 512 514 10.1126/science.283.5401.512 9915692

[B8] Jun H WonBong C Controlled growth and electrical characterization of bent single-walled carbon nanotubes Nanotechnology 2008 19 505601 10.1088/0957-4484/19/50/505601 19942773

[B9] Chhowalla M Teo KBK Ducati C Rupesinghe NL Amaratunga GAJ Ferrari AC Roy D Robertson J Milne WI Growth process conditions of vertically aligned carbon nanotubes using plasma enhanced chemical vapor deposition J Appl Phys 2001 90 5308 5317 10.1063/1.1410322

[B10] Kumar A Pushparaj VL Kar S Nalamasu O Ajayan PM Baskaran R Contact transfer of aligned carbon nanotube arrays onto conducting substrates Appl Phys Letters 2006 89 163120 163123 10.1063/1.2356899

[B11] Yung KP Wei J Tay BK Formation and assembly of carbon nanotube bumps for interconnection applications Diam Relat Mater 2009 18 1109 1113 10.1016/j.diamond.2009.02.022

[B12] Yap CC Tan D Brun C Li H Teo EHT Baillargeat D Tay BK Impact of the CNT growth process on gold metallization dedicated to RF interconnect applications Intl J Microwave Wireless Technol 2010 2 463 469 10.1017/S1759078710000681

[B13] Fan SH Chan YC Effect of misalignment on electrical characteristics of ACF joints for flip chip on flex applications Microelectron Reliability 2002 42 1081 1090 10.1016/S0026-2714(02)00069-0

[B14] Glickman M Tseng P Harrison J Goldberg IB Johnson P Smeys P Niblock T Judy JW CMOS-compatible back-end process for in-place actuating ferromagnetic MEMS Solid-State Sensors, Actuators and Microsystems Conference, 2009 TRANSDUCERS 2009 International 21-25 June 2009; Denver 2009 New York: IEEE 248 251

[B15] Hofmann S Ducati C Robertson J Kleinsorge B Low-temperature growth of carbon nanotubes by plasma-enhanced chemical vapor deposition Appl Phys Letters 2003 83 135 10.1063/1.1589187

[B16] McEuen PL Fuhrer MS Hongkun P Single-walled carbon nanotube electronics Nanotechnol, IEEE Trans on 2002 1 78 85 10.1109/TNANO.2002.1005429

[B17] Jeong G-H Olofsson N Falk LKL Campbell EEB Effect of catalyst pattern geometry on the growth of vertically aligned carbon nanotube arrays Carbon 2009 47 696 704 10.1016/j.carbon.2008.11.003

[B18] Berber S Kwon Y-K Tomanek D Bonding and energy dissipation in a nanohook assembly Phys Rev Letters 2003 91 165503 10.1103/PhysRevLett.91.165503 14611412

[B19] Nihei M Kawabata A Kondo D Horibe M Sato S Awano Y Electrical properties of carbon nanotube bundles for future via interconnects Jpn J Appl Phys 2005 44 1626 10.1143/JJAP.44.1626

[B20] Santini CA Volodin A Van Haesendonck C De Gendt S Groeseneken G Vereecken PM Carbon nanotube-carbon nanotube contacts as an alternative towards low resistance horizontal interconnects Carbon 2011 49 4004 4012 10.1016/j.carbon.2011.05.041

[B21] Fuhrer MS Nygård J Shih L Forero M Yoon Y-G Mazzoni MSC Hyoung Joon C Jisoon I Louie SG Zettl A McEuen PL Crossed nanotube junctions Science 2000 288 494 497 10.1126/science.288.5465.494 10775104

[B22] Li H Loke WK Zhang Q Yoon SF Physical device modeling of carbon nanotube/GaAs photovoltaic cells Appl Phys Letters 2010 96 043501 043503 10.1063/1.3293452

[B23] Bachtold A Strunk C Salvetat J-P Bonard J-M Forro L Nussbaumer T Schonenberger C Aharonov-Bohm oscillations in carbon nanotubes Nature 1999 397 673 675 10.1038/17755

[B24] Li H Zhang Q Li J Interpretation of coulomb oscillations in carbon-nanotube-based field-effect transistors Phys Rev B 2006 73 235431

